# Phenotypic association among performance, feed efficiency and methane emission traits in Nellore cattle

**DOI:** 10.1371/journal.pone.0257964

**Published:** 2021-10-14

**Authors:** Leandro Sannomiya Sakamoto, Luana Lelis Souza, Sarah Bernardes Gianvecchio, Matheus Henrique Vargas de Oliveira, Josineudson Augusto II de Vasconcelos Silva, Roberta Carrilho Canesin, Renata Helena Branco, Melissa Baccan, Alexandre Berndt, Lucia Galvão de Albuquerque, Maria Eugênia Zerlotti Mercadante

**Affiliations:** 1 Institute of Animal Science, Beef Cattle Research Center, Sertãozinho, SP, Brazil; 2 São Paulo State University (Unesp), School of Agricultural and Veterinarian Sciences, Jaboticabal, SP, Brazil; 3 São Paulo State University (Unesp), School of Veterinary Medicine and Animal Science (FMVZ), Botucatu, São Paulo, Brazil; 4 Embrapa Environment, Jaguariúna, SP, Brazil; 5 Embrapa Southeast Livestock, São Carlos, SP, Brazil; Universidade Federal de Viçosa, BRAZIL

## Abstract

Enteric methane (CH_4_) emissions are a natural process in ruminants and can result in up to 12% of energy losses. Hence, decreasing enteric CH_4_ production constitutes an important step towards improving the feed efficiency of Brazilian cattle herds. The aim of this study was to evaluate the relationship between performance, residual feed intake (RFI), and enteric CH_4_ emission in growing Nellore cattle (*Bos indicus*). Performance, RFI and CH_4_ emission data were obtained from 489 animals participating in selection programs (mid-test age and body weight: 414±159 days and 356±135 kg, respectively) that were evaluated in 12 performance tests carried out in individual pens (n = 95) or collective paddocks (n = 394) equipped with electronic feed bunks. The sulfur hexafluoride tracer gas technique was used to measure daily CH_4_ emissions. The following variables were estimated: CH_4_ emission rate (g/day), residual methane emission and emission expressed per mid-test body weight, metabolic body weight, dry matter intake (CH_4_/DMI), average daily gain, and ingested gross energy (CH_4_/GE). Animals classified as negative RFI (RFI<0), i.e., more efficient animals, consumed less dry matter (*P <0*.*0001*) and emitted less g CH_4_/day (*P = 0*.*0022*) than positive RFI animals (RFI>0). Nonetheless, more efficient animals emitted more CH_4_/DMI and CH_4_/GE (*P < 0*.*0001*), suggesting that the difference in daily intake between animals is a determinant factor for the difference in daily enteric CH_4_ emissions. In addition, animals classified as negative RFI emitted less CH_4_ per kg mid-test weight and metabolic weight (*P = 0*.*0096* and *P = 0*.*0033*, respectively), i.e., most efficient animals could emit less CH_4_ per kg of carcass. In conclusion, more efficient animals produced less methane when expressed as g/day and per kg mid-test weight than less efficient animals, suggesting lower emissions per kg of carcass produced. However, it is not possible to state that feed efficiency has a direct effect on enteric CH_4_ emissions since emissions per kg of consumed dry matter and the percentage of gross energy lost as CH_4_ are higher for more efficient animals.

## Introduction

Enteric methane (CH_4_) emission is a natural process in ruminants that can result in losses of 2 to 12% of the total energy consumed by the animal [1]. The variation is the result of some factors such as chemical composition of the diet, intake level [2], and even genetic [3] and metagenomic [4].

Residual feed intake (RFI) has been used as a selection criterion in beef cattle in order to increase individual feed efficiency [5,6]. Most efficient animals (negative RFI) have a significant economic advantage as they consume less dry matter than expected for their weight and weight gain [7]. Consequently, the use of negative RFI animals has the potential to significantly reduce meat production costs.

Generally, the higher the dry matter intake (DMI), the higher the daily enteric emissions of CH_4_ since a larger amount of substrate will be available for fermentation in the rumen and consequently more hydrogen will be available for methanogenesis [8,9]. Therefore, the use of more efficient animals may reduce enteric CH_4_ emissions proportionally to the lower feed intake [10]. However, it is unclear whether the differences in enteric CH_4_ emissions are due to the variation in digestive efficiency between negative and positive RFI animals or simply the result of from the lower DMI associated with negative RFI animals [11,12].

Inconsistencies still exist regarding the relationship between feed efficiency (RFI and FC) and enteric CH_4_ emission by cattle. Studies have shown this correlation is positive and favorable in the case of highly digestible diets [10,13,14], while the phenotypic relationship between feed efficiency and enteric CH_4_ emission is zero or even negative and unfavorable in diets with low digestibility [14–17], suggesting that individual enteric CH_4_ emission may even increase with the improvement of feed efficiency. Furthermore, few studies have investigated *Bos indicus* animals receiving high roughage diets [16,18,19] and there is a lack of studies involving a large number of zebuine animals. The aim of the present study was to evaluate the relationship among performance, feed efficiency and enteric CH_4_ emission traits in Nellore cattle (*Bos indicus*). The hypothesis was that the use of animals with low DMI and similar ADG could be a strategy to reduce greenhouse gases emissions in the beef production system.

## Materials and methods

### Location and animals

The data were collected in 2011, 2012, 2018, 2019 and 2020 in Sertãozinho-SP, Brazil, as well as in Botucatu-SP, Brazil, in 2019. Performance, feed efficiency and enteric CH_4_ emission data were obtained from 489 Nellore animals evaluated in performance tests. This study was carried out in strict accordance with the recommendations in the Guidelines for Animal Welfare and Humane Slaughter (São Paulo State, Law Number 11.977). The protocol was approved by the Committee on the Ethics of Animal Experiments of the Institute of Animal Science (Protocol Number 278–19), Nova Odessa-SP, Brazil.

### Treatments and management

The performance tests had an average duration of 76.5 ± 12 days preceded by 28 days of adaptation [5]. The animals started the test at 376 ± 164 days of age, from June to December of each year, and were kept in individual pens (n = 95) or collective paddocks equipped with electronic feed bunks (GrowSafe®, Airdrie-AB, Canada; or Intergado®, Contagem-MG, Brazil) for automated recording of individual daily feed intake (n = 394), with *ad libitum* access to diet and water. The animals were weighed at the beginning and end of the test after fasting for 14 h, or at predetermined intervals without previous fasting (Table 1).

**Table 1:** Description of test groups for evaluating the association among performance, feed efficiency and enteric methane emission traits of Nellore (*Bos indicus*).

Group	Year	Sex category	Days in test	Facility	Collector container^3^	Capsule emission (mg SF_6_/day)	No. of animals	Initial age (days)	Initial weight (kg)	No. of weight recordings
1	2011	Heifers	83	Individual pen	Canister	1.623 ± 0.08	23	294 ± 26	219 ± 28	4
2	2011	Bulls	71	Individual pen	Canister	1.405 ± 0.05	23	268 ± 24	254 ± 34	19
3	2012	Bulls	90	Individual pen	Canister	2.334 ± 0.19	24	264 ± 23	229 ± 34	13
4	2012	Heifers	85	Individual pen	Canister	1.938 ± 0.16	25	325 ± 26	261 ± 28	14
5	2018	Bulls	83	GrowSafe®	Cylinder	3.119 ± 0.27	34	347 ± 28	270 ± 46	6
6	2018	Bulls	83	GrowSafe®	Cylinder	3.145 ± 0.23	36	354 ± 25	275 ± 43	6
7	2019	Bulls	83	GrowSafe®	Cylinder	4.549 ± 0.30	60	249 ± 31	224 ± 33	6
8	2019	Bulls	56	Intergado®	Cylinder	3.471 ± 0.17	58	647 ± 36	465 ± 39	2
9	2019	Bulls	56	Intergado®	Cylinder	3.062 ± 0.09	58	667 ± 35	573 ± 48	2
10	2019	Bulls	83	GrowSafe®	Cylinder	2.471 ± 0.15	62	329 ± 24	285 ± 49	7
11	2020	Bulls	83	GrowSafe®	Cylinder	2.621 ± 0.35	42	237 ± 24	226 ± 42	7
12	2020	Bulls	83	GrowSafe®	Cylinder	2.656 ± 0.34	44	239 ± 22	221 ± 33	7

In each test, the animals were fed a single diet that differed among the years. The diets during the performance tests consisted of silage (corn or sorghum), *Brachiaria* hay, sugar cane bagasse, meal (cottonseed, soybean or peanut), corn (ground or wet grain), citrus pulp, mineral premix, salt, ammonium sulfate, and urea (Table 2).

**Table 2:** Percentage of ingredients and nutrient composition of diets offered to the animals during the performance test according each test group.

Ingredients (% DM)	Year of performance test					
	2011	2012	2018	2019	2019	2020
Corn silage	-	53.6	54.0	-	27.6	60.0
Sorghum silage	-	-	-	60.0	-	-
*Brachiaria* hay	44.5	10.1	-	-	-	-
Sugar cane bagasse	-	-	10.2	-	4.89	-
Cottonseed meal	21.4	-	-	-	-	-
Soybean meal	-	11.6	11.7	13.0	-	13.0
Peanut meal	-	-	-	-	8.01	-
Ground corn	32.2	21.7	21.9	25.0	-	25.0
Wet corn	-	-	-	-	44.6	-
Citrus pulp	-	-	-	-	11.9	-
Mineral premix	-	-	-	-	1.78	-
Salt	1.45	2.28	1.70	1.75	-	1.75
Ammonium sulfate	-	0.072	-	-	-	-
Urea	0.45	0.648	0.49	0.25	1.16	0.25
Forage to concentrate ration	65:45	65:45	60:40	60:40	50:50	60:40
Nutrients						
Dry matter, %	87.4	54.4	60.5	52.4	60.0	52.9
Crude protein, % DM	11.3	13.9	10.6	11.2	15.6	10.6
Ash, % DM	3.74	-	3.69	4.63	-	4.08
Ether extract, % DM	2.84	1.90	1.78	2.13	3.20	3.29
Neutral detergent fiber, % DM	50.0	50.2	48.1	40.6	26.9	35.6
Acid detergent fiber, % DM	31.0	22.9	30.7	24.4	-	21.3
Gross energy, Mcal/kg	4.09	4.16	3.73	3.77	4.11	4.47
Non-fiber carbohydrates, DM%	32.1	34.0	35.8	41.5	54.0	46.4
Total digestible nutrients^1^, DM%	70.5	70.2	65.9	70.2	77.0	75.1

^1^Values calculated using the equation of Weiss [20]. DM: Dry matter. The diets were formulated for 0.800 kg/day in 2011 and 2012, for 1.200 kg/day in 2018, 2019 (60:40), and 2020, and for 1.700 kg/day in 2019 (50:50) [21].

After pre-drying (55 ± 5°C for 72 h), diet samples were ground in a Willey-type mill (R-TE-650 model, Tecnal Equipamentos Científicos, Piracicaba, São Paulo, Brazil) to pass a 1-mm screen and analyzed for dry matter (method 934.01), ash (method 942.05) and ether extract (method 920.39) contents following the AOAC [22] guidelines. The contents of neutral detergent fiber (NDF) and acid detergent fiber (ADF) were determined by the methodology of Mertens [23] using a Tecnal fiber analyzer (TE-149, Piracicaba, São Paulo, Brazil) using α-amylase and without sodium sulphite. The NDF and ADF were expressed exclusive of ash. The determination of crude protein (method 990.03) was performed by the Dumas method [24] based on the release of nitrogen by combustion at high temperature in pure oxygen in DUMATHERM® analyzer. Total carbohydrates were calculated according to the methodology described by Sniffen et al. [25]: CHOT = 100 - (CP + EE + MM); and non-fiber carbohydrates were obtained by subtracting the NDF. The gross energy (GE) determinations were performed in an adiabatic calorimetric pump of the brand IKA WERKE Model C5003 (Parr Instrument Company, Illinois, USA).

The amount of feed was adjusted weekly to guarantee daily leftovers of 5 to 10% of the total amount supplied in order to ensure *ad libitum* intake. The troughs were cleaned and leftovers were removed and discarded three times per week. Intake records were discarded when there were no feed leftovers and in the case of evidence of malfunctioning of the electronic measurement devices. Weekly samples of the ingredients were obtained for determination of the dry matter content of the diet.

### Animal performance and feed efficiency

The following traits were calculated as described by Grion et al. [5] and Ceacero et al. [6]: DMI, average daily gain (ADG), metabolic body weight (BW^0.75^), RFI, and feed conversion (FC). The DMI was obtained as the mean of all valid days during the period. The ADG was estimated by the linear regression coefficient of weights on days in test (DIT) according to the equation: yi = α + β x DITi + Ɛi, where yi = weight of the animal in the *i*^th^ observation; α = intercept representing the initial weight of the animal; β = linear regression coefficient representing ADG; DITi = days in test in the *i*^th^ observation; Ɛi = random error associated with each observation. The BW^0.75^ was obtained as follows: BW^0.75^ = (BWi + (0.5 DIT x ADG))^0.75^, where BWi = initial body weight and DIT = days in test.

The RFI was calculated as the difference between observed and expected DMI, which was estimated by multiple regression of DMI on ADG and BW^0.75^ within the test group [i = 1,…, 12; formed by year of birth, sex (48 females and 441 intact males), facility, site)], using the GLM procedure (SAS Inst., Inc., Cary, NC). The FC was obtained as the ratio between DMI and ADG. Mean residual gain was calculated as the difference between observed and expected ADG, which was estimated by multiple regression of ADG on DMI and BW^0.75^ within the test group, using the same procedure as described above.

### Ruminal methane measurement

The modified sulfur hexafluoride (SF_6_) tracer gas technique described by Deighton et al. [26] was used for methane collection. The technique uses a permeation tube or capsule administered to the animal and deposited in the rumen. These capsules were calibrated and prepared specifically for each sampling year. The mean SF_6_ gas emission of the capsules used during each sampling period was similar, with minimal variation in mg/day (Table 1).

**Fig 1:**
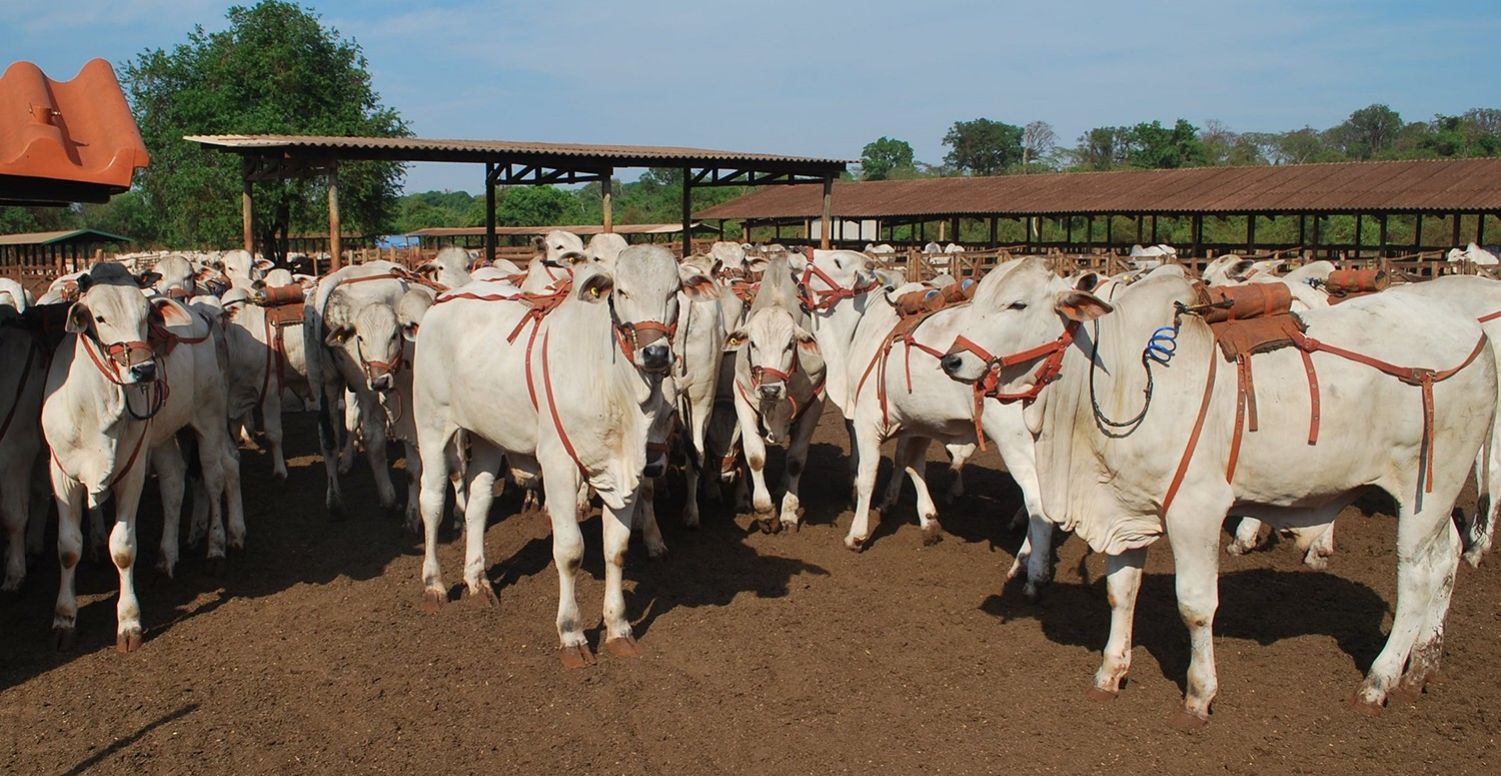
Animals, in a test group, with the apparatus (halter, saddle and cylinder) for methane collection by sulfur hexafluoride (SF_6_) tracer gas technique.

Before the beginning of the sampling period, the animals were adapted to the sampling apparatus for at least seven days. Methane gas was collected for five consecutive days, with the evacuated sampling polyvinyl chloride canisters (n = 95 animals) [16,18] or stainless-steel cylinder (n = 394 animals) being changed every 24 hours (Fig 1). The gas expelled through the mouth and nostrils of the animal was aspirated under vacuum with a capillary tube fixed in a halter and connected to the collector container, which was attached to the neck of the animal (polyvinyl chloride canister) or to a saddle on the back of the animal (stainless-steel cylinder) (Table 1). Collector tubes were kept in the same environment as the animals to measure background concentrations of CH_4_ and SF_6_ during the sampling period. After each sampling period, the collectors were sent for gas chromatography analysis and their content was diluted with pure nitrogen to determine the quantities of SF_6_ and CH_4_ gases. The background concentrations of CH_4_ and SF_6_ measured by chromatography were subtracted from the concentrations found in the evacuated sampling containers of the animals.

There was a total of 12 CH_4_ sampling periods (Table 1). Of the 489 animals evaluated, samples from 481 animals could be used. The losses were due to problems with the capsules. The sampling periods were September 2011; October and December 2012; November and December 2018; June, August and October 2019, and August 2020.

A gas chromatograph (HP6890, Agilent, Wilmington, Delaware, USA) was used for the analysis of CH_4_ (ppm, parts per million) and SF_6_ (ppt, parts per trillion). The concentrations of CH_4_ and SF_6_ collected in the evacuated sampling containers were determined with a flame ionization detector at 280°C (HP-Plot Al_2_O_3_ M column, 30 m length × 0.53 mm i.d. × 15 μm film thickness) and an electron capture detector at 300°C (HP-Plot MoleSieve column, 30 m length × 0.53 mm i.d. × 25 μm film thickness), respectively, with two loops of 0.5 cm^3^ maintained at 80°C attached to 2 six-way valves. Chromatography analysis was carried out immediately after the end of the field sampling periods, which allowed the reuse of the evacuated containers in the subsequent sampling period.

### Methane-related variables

Daily CH_4_ emission (g/day) of each animal was obtained as the arithmetic mean of emissions on five consecutive sampling days. Enteric methane emission was also expressed as: CH_4_ emission expressed per DMI (CH_4_/DMI, g/kg), ADG (CH_4_/ADG, g/kg), mid-test body weight (CH_4_/MBW, g/kg) and BW^0.75^ (CH_4_/BW^0.75^, g/kg), residual CH_4_ emission (observed CH_4_ –predicted CH_4_ by regression of CH_4_ on DMI as described by Donoghue et al. [3]), and CH_4_ emission expressed per gross energy intake (CH_4_ Mcal/100 Mcal GE, as described by IPCC [27]).

### Statistical analysis

The animals were classified as negative RFI (RFI<0) or positive RFI (RFI>0). The variables were analyzed using the MIXED procedure (SAS Inst., Inc., Cary, NC), fitting a model that included the fixed effect of RFI class (i = 1, 2), age of animal at the start of the performance test as covariate (linear effects), and the random effects of test group (i = 1,…, 12), in addition to the residual random effect. The relationships of CH_4_ (g/day) with DMI, ADG and MBW were explored by Pearson’s correlation and regression analyses using the CORR and GML procedures (SAS Inst., Inc., Cary, NC). The regression model for CH_4_ (g/day) included the linear effect of DMI or ADG or MBW as covariate and the random effects of test group and residual. Statistical significance was declared when P<0.05.

## Results

The mean weights (initial, mid-test and metabolic) or ADG did not differ between animals classified as negative and positive RFI (Table 3). The mean RFI was -0.556 and 0.565 kg DM/day for negative and positive RFI animals, respectively, showing a difference in DMI of 1.16 kg/day between the most and least efficient animals. Animals classified as negative RFI consumed on average 13% less DM than animals classified as positive RFI; consequently, FC and residual ADG higher for more efficient animals. There was a 5% reduction of CH_4_ emission (g/day) in negative RFI animals compared to animals with positive RFI. In addition, despite a similar performance, more efficient animals emitted less methane expressed as g CH_4_/kg MBW and g CH_4_/kg BW^0.75^. Conversely, lower CH_4_ emission in relation to DMI (g CH_4_/kg DMI), lower residual CH_4_ emission and a lower percentage of GE lost as CH_4_ were observed in positive RFI animals compared to animals with negative RFI (Table 3).

**Table 3:** Mean values of performance, feed efficiency and enteric methane emission traits according to residual feed intake class of Nellore (*Bos indicus*).

Trait	N	Negative RFI (n = 246)	Positive RFI (n = 243)	SEM	P
Initial age (days)	489	390	389	44.0	0.5353
Initial body weight (kg)	489	317	317	34.2	0.8675
Mid-test body weight (kg)	489	353	354	11.2	0.8498
Dry matter intake (kg/day)	489	7.405	8.550	0.23	<0.0001
Average daily gain (kg/day)	489	1.228	1.237	0.07	0.7121
Metabolic body weight (kg)	489	79.7	79.8	1.59	0.8937
RFI (kg/day)	489	-0.556	0.565	0.03	<0.0001
Feed conversion (kg/kg)	489	6.695	7.764	0.453	<0.0001
Residual average daily gain (kg/day)	489	0.066	-0.064	0.014	<0.0001
CH_4_ (g/day)	481	179.7	189.8	10.1	0.0022
CH_4_/DMI (g/kg/day)	481	23.46	21.34	1.09	<0.0001
CH_4_/ADG (g/kg/day)	481	169.3	175.2	16.2	0.0724
CH_4_/MBW (g/kg)	481	0.529	0.548	0.03	0.0096
CH_4_/BW^0.75^ (g/kg)	481	2.259	2.353	0.14	0.0033
CH_4_Res (g/day)	481	4.811	-4.953	1.95	0.0004
CH_4_/GE (%GE)	481	7.78	7.08	0.41	<0.0001

RFI: Residual feed intake; SEM: Standard error of the mean; CH_4_: Enteric methane emission; CH_4_/DMI: CH_4_ emission expressed per dry matter intake; CH_4_/ADG: CH_4_ emission expressed per average daily gain, CH_4_/MBW: CH_4_ emission expressed per mid-test body weight; CH_4_/BW^0.75^ = CH_4_ emission expressed per metabolic body weight; CH_4_Res: Residual CH_4_ emission; CH_4_/GE: % consumed gross energy lost as CH_4_.

**Fig 2:**
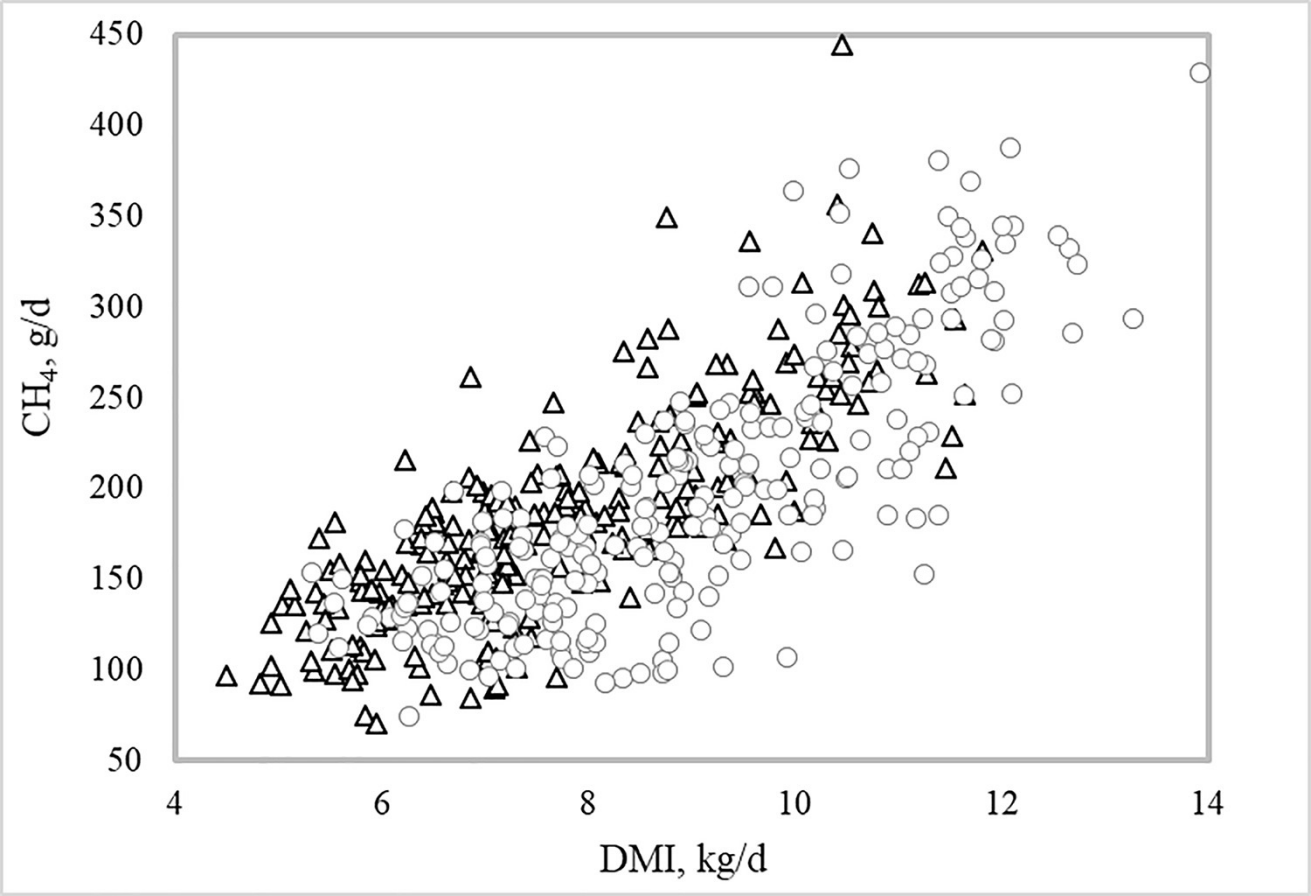
**Relationship between enteric methane (CH**_**4**_**) emissions and dry matter intake (DMI) of Nellore bulls and heifers classified as negative (triangle) or positive (circle) residual feed intake (RFI).** The general linear regression equation of CH_4_ on DMI was: y = 39.0 (±14.6) + 17.5(±1.23)x + residual.

**Fig 3:**
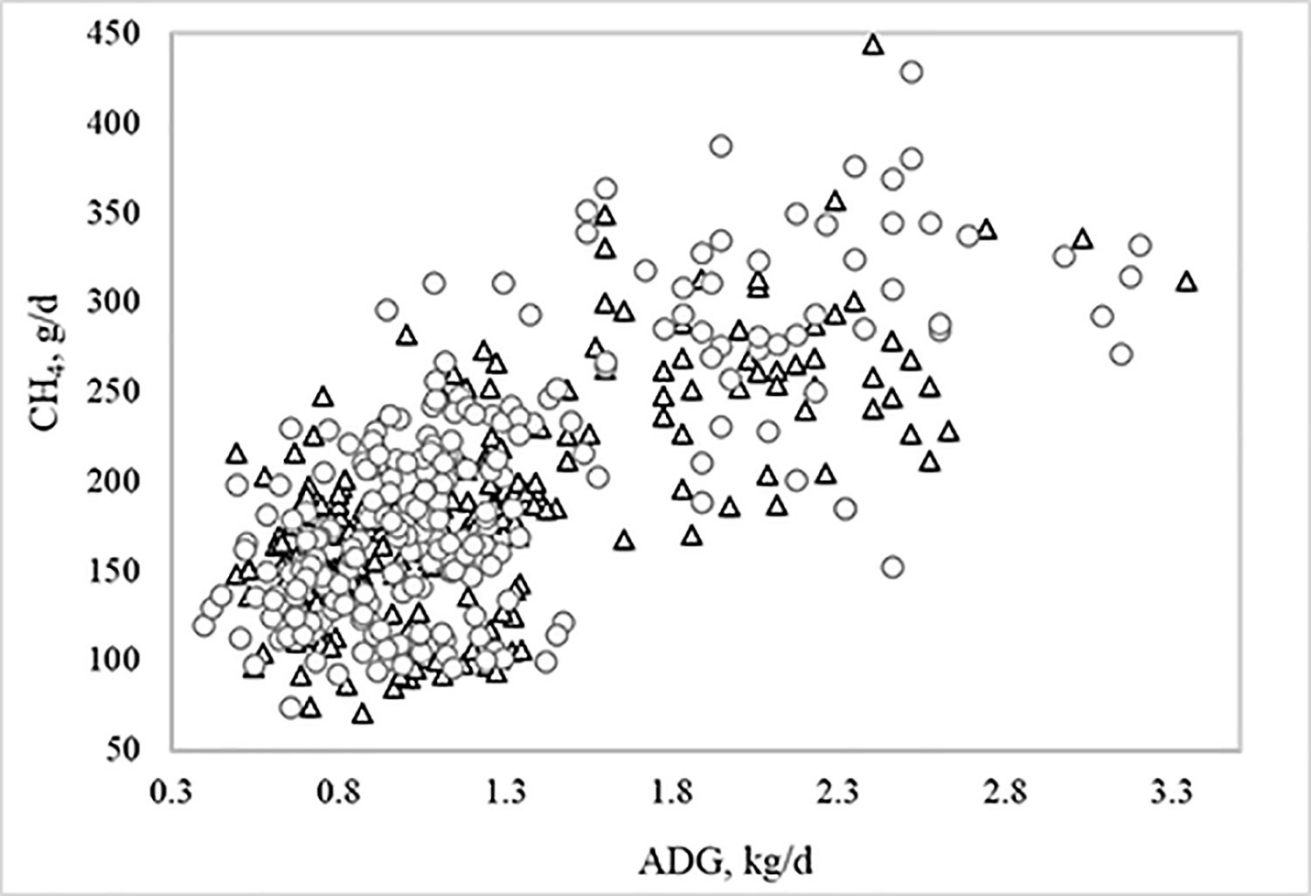
**Relationship between enteric methane (CH**_**4**_**) emissions and average daily gain (ADG) of Nellore bulls and heifers classified as negative (triangle) or positive (circle) residual feed intake (RFI).** The general linear regression equation of CH_4_ on ADG was: y = 112(±13.7) + 58.0(±5.31)x + residual.

**Fig 4:**
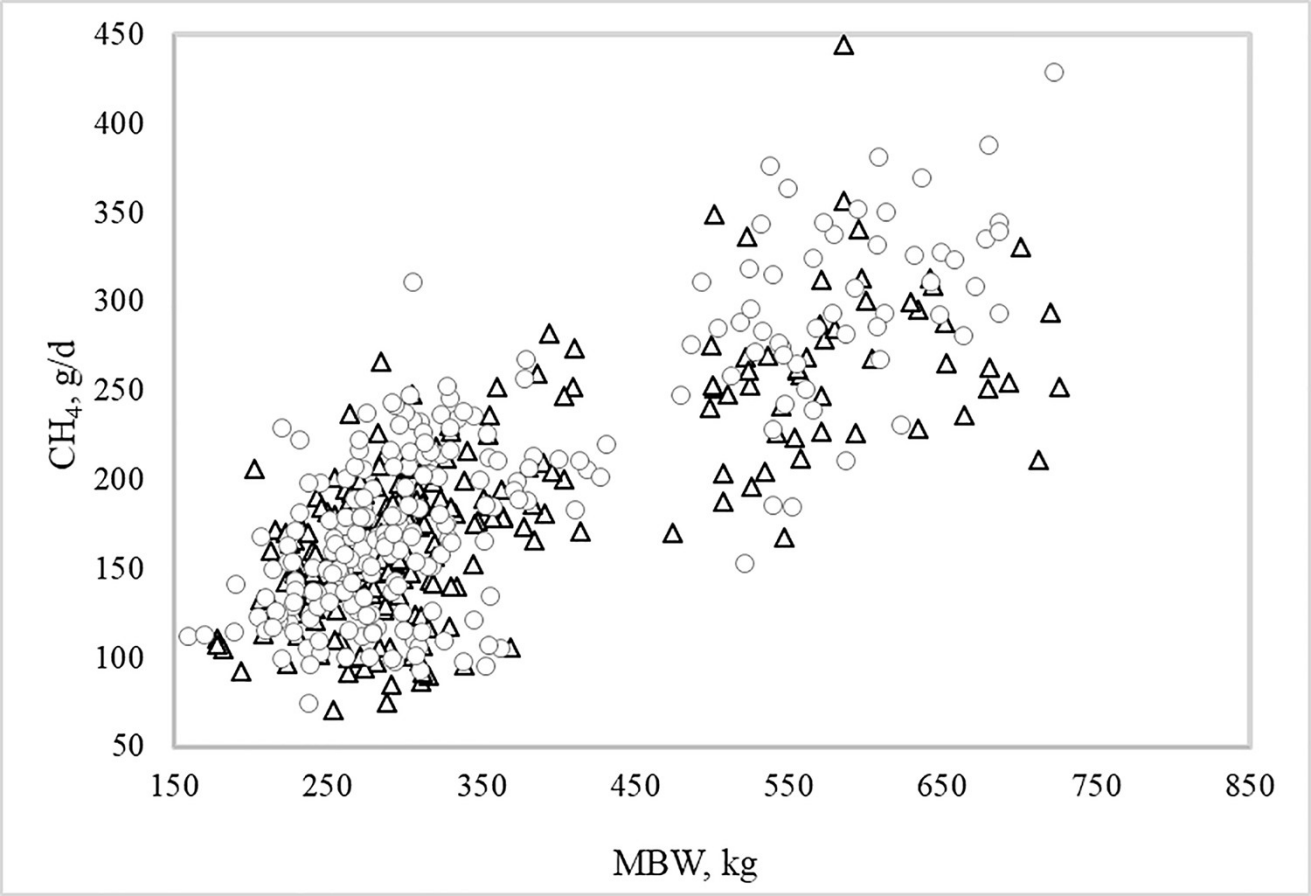
**Relationship between enteric methane (CH**_**4**_**) emissions and mid-test body weight (MBW) of Nellore bulls and heifers classified as negative (triangle) or positive (circle) residual feed intake (RFI).** The general linear regression equation of CH_4_ on MBW was: y = 35,7(±13.9) + 0.43(±0.030)x + residual.

The simple correlation coefficients of CH_4_ (g/day) with DMI, ADG and MBW were 0.77, 0.70, and 0.78 (*P<0*.*0001*), respectively. Scatter plots of CH_4_ (g/day) with DMI, ADG and MBW and their respective regression equations are shown in Figs 2–4. For each kg of DMI the animals emitted on average 17.5 g CH_4_/day, for each kg of ADG the animals emitted on average 58.0 g CH_4_/day, and for each kg of MBW the animals emitted 36.0 g CH_4_/day. The regression equations of CH_4_ on DMI, ADG and MBW within RFI class differed from one another (*P = 0*.*001*), accompanying the results shown in Table 3.

## Discussion

In general, studies investigating the relationship between feed efficiency and enteric CH_4_ emissions in cattle did not include a large number of animals, mainly because of the difficulty in measuring individual enteric CH_4_ emissions in the animals, and the ones that did were conducted on *Bos taurus* (Table 4). In contrast, the present study evaluated 489 *Bos indicus* animals and enteric CH_4_ was measured individually by the SF_6_ tracer gas technique.

**Table 4:** Studies in the literature showing the relationship between residual feed intake classes and enteric methane emission.

Reference	N	Sex category	Cattle breed	Measurement technique	CH_4_ (g/day)		*P*	CH_4_/DMI (g/kg/day)		*P*
					RFI-	RFI+		RFI-	RFI+	
Nkrumah et al. [13]	19	Steers	Continental x British	Indirect calorimetry	135	180	*<0*.*05*	14.0	15.5	*-*
Hegarty et al. [10]	20	Steers	Angus	SF_6_	142	190	*0*.*01*	16.3	14.7	*0*.*37*
Jones et al. [14]	25 48	Pregnant cows Cows	Angus	OP-FTIR	133 182	125 227	*-* *<0*.*05*	13.0 13.9	11.7 16.2	*-* *-*
Fitzsimons et al. [28]	14	Heifers	Simmental	SF_6_	260	297	*0*.*04*	38.0	36.0	*0*.*52*
Sharma et al. [29]	6	Calves	Sahiwal	SF_6_	58.7	65.6	*<0*.*05*	15.3	18.9	*<0*.*05*
McDonnell et al. [30]	28	Heifers	Limousin x Friesian	SF_6_	156	146	*0*.*11*	22.4	20.2	*0*.*034*
Alemu et al. [31]	16	Heifers	Crossbred	GreenFeed Respirometry chamber	203 156	222 165	*0*.*02* *0*.*40*	27.7 26.5	28.5 26.5	*0*.*25* *0*.*99*
Dini et al. [32]	16	Steers	Hereford	SF_6_	194	265	*0*.*009*	20.3	28.1	*0*.*021*
Flay et al. [33]	56	Heifers	Jersey/Holstein-Friesian	GreenFeed	253	256	*0*.*60*	22.7	20.7	*<0*.*01*
Manafiazar et al. [34]	314 139	Heifers Cows	Crossbred	GreenFeed	180 233	184 241	*0*.*001* *<0*.*001*	24.1 21.1	22.7 19.2	*<0*.*001* *<0*.*001*
Batalha et al. [19]	24	Bulls	Nellore	SF_6_	235	249	*0*.*365*	25.3	26.2	*0*.*389*

SF_6_: SF_6_ tracer gas technique; OP-FTIR: Open-path Fourier transform infrared spectroscopy; CH_4_: Methane emission; RFI-: Negative residual feed intake.; RFI+: Positive residual feed intake.

Greater reductions in enteric CH_4_ emissions (15–30%) were reported in the literature for more efficient taurine animals [10,13,32] compared to the reduction of approximately 5% in the emission of zebuine animals classified as negative RFI in the present study (Table 3). Results similar to those of the present study were observed in crossbred taurine heifers and cows classified as more efficient, with a daily CH_4_ reduction of 2.5% and 3.7%, respectively, compared to less efficient animals [34]. Lower enteric CH_4_ emissions were also reported for negative RFI Angus cows compared to positive RFI cows grazing on high-quality pasture [14]. However, there was no difference in CH_4_ emissions (g/day) for animals grazing a pasture of low nutritional quality [14]. On the other hand, Freetly and Brown-Brandl [15], Velazco et al. [17], Flay et al. [33] and Batalha et al. [19] found no differences in CH_4_ emissions (g/day) between more and less efficient animals.

The difference in the DMI of the animals might be responsible for the differences in daily enteric CH_4_ emissions between RFI classes [9,35], which would explain the results found in the present study (Table 3). Given that they have the same body weight, same weight gain and same amount of body fat, negative RFI animals tend to emit less CH_4_ per day because of lower DMI [34]. Some studies evaluating CH_4_ emission in animals classified as negative and positive RFI reported differences in emissions per kg of DMI, although they found no differences in CH_4_ production [30,33]. However, other studies reported lower CH_4_ emissions and lower CH_4_ emission per DMI in negative RFI animals [29,32]. One explanation would be a lower particle passage rate in the rumen due to differences in feeding behavior since more efficient animals spend less time feeding and therefore exhibit a higher feeding rate than positive RFI animals [19,32]. Residual feed intake is an intrinsic trait of the individual that reflects maintenance requirements [36]; thus, another explanation for the lower CH_4_ emissions would be a lower energy intake of negative RFI animals compared to positive RFI animals [29], since CH_4_ emission is positively associated with energy intake and differences in digestibility, CH_4_ emissions, heat production and energy retention are the main factors responsible for the variation of RFI between animals [13].

Lower or equal CH_4_ emission (g/day), but higher emission per kg of DMI (CH_4_/DMI) and a higher percentage of gross energy lost as CH_4_, in negative RFI animals compared to positive RFI were observed in the present study and have also been reported by other authors [30,33,34]. The reasons for these differences in CH_4_/DMI and CH_4_/GE between RFI classes are still unclear. Possible explanation is an increase in rumen organic matter degradation with consequent increase in H_2_ ions availability for methanogenesis in negative RFI animals [30]. Nellore animals classified based on RFI from the same contemporary groups differed in their nutrient digestive capacity [19,37,38]. Although Magnani et al. [37] and Bonilha et al. [38] demonstrated higher digestibility of dry matter (8%), neutral detergent fiber (13 to 19%) and acid detergent fiber (11%) in positive RFI animals compared to negative RFI animals, Batalha et al. [19] found lower digestibility (4.7% to 9%) of dry matter, neutral detergent fiber and acid detergent fiber, as well as of crude protein.

The lower CH_4_ emissions per kg of live and metabolic weight observed in animals classified as negative RFI (*P = 0*.*0096* and *P = 0*.*0033* for CH_4_/MBW and CH_4_/BW^0.75^, respectively) were similar to the results reported by Nkrumah et al. [13] and Fitzsimons et al. [28]. These findings indicate that, regardless of the effects of DMI, potential selection of cattle for RFI and reduced enteric CH_4_ emission is possible [16,28]. The strong relationship between CH_4_ and DMI and the divergent results reported in the literature for RFI and CH_4_ emissions underscore the lack of evidence of a direct effect of RFI on enteric methane emission [35].

Considering the number of animals evaluated in this study and the fact that negative and positive RFI animals were compared (and not only extreme animals), it is possible to state that CH_4_ emissions per kg of live body weight were different, with lower values in more efficient animals. This confirms the hypothesis that negative RFI animals emit less CH_4_ per day or per kg of live body weight or per kg of ADG and, furthermore, these animals have strong potential to emit less CH_4_ per kg of carcass. Considering that live body weight in the present study is the yearling weight, and the evidence of high genetic (0.55 to 0.89) and phenotypic (0.67 to 0.72) correlation between yearling weight and carcass weight [39–41], live body weight is a real indicator of the carcass weight.

Differences in the number of methanogenics in the rumen between more and less efficient animals may explain the differences observed in the intensity of CH_4_ emission, regardless of the diet supplied [42]. In fact, Lopes et al. [43] and Andrade et al. [44] reported differences in the microbial composition of fecal samples between Nellore animals classified as negative and positive RFI. However, these differences in microbial populations between negative and positive RFI cattle may be due to differences in the ruminal passage rate and digestion as a result of different DMI levels [45]. Another approach to explain the variation in CH_4_ emission between more and less efficient animals independent of DMI would be to identify differences in the efficiency of feed utilization, such as nutrient absorption, appetite regulation, and cell metabolism [35].

In conclusion, more efficient animals emit less CH_4_ expressed as g/day and per kg of live body weight than less efficient animals, suggesting lower emission per kg of carcass. However, it is not possible to state that RFI has a direct effect on enteric CH_4_ emissions since emission per kg of consumed dry matter and the percentage of gross energy lost as CH_4_ were greater for negative RFI animals.

## Supporting information

S1 Table Description of test groups for evaluating the association among performance, feed efficiency and enteric methane emission traits of Nellore (*Bos indicus*). (DOCX)

S2 Table Percentage of ingredients and nutrient composition of diets offered to the animals during the performance test according each test group. (DOCX)

S3 Table Mean values of performance, feed efficiency and enteric methane emission traits according to residual feed intake class of Nellore (*Bos indicus*). (DOCX)

S4 Table Studies in the literature showing the relationship between residual feed intake classes and enteric methane emission. (DOCX)
