# Supplementary material for: Phenotypic association among performance, feed efficiency and methane emission traits in Nellore cattle
Source: PLoS One. 2021 Oct 14;16(10):e0257964. doi: 10.1371/journal.pone.0257964 (PMC8516271; doi:10.1371/journal.pone.0257964)
Supplement: S1 Table — (DOCX) [file pone.0257964.s001.docx]

**S1 Table.** **Description of test groups for evaluating the association among performance, feed efficiency and enteric methane emission traits of Nellore (*Bos indicus*)**

| Group | Year | Sex category | Days in test | Facility | Collector container^3^ | Capsule emission  (mg SF_6_/day) | No. of animals | Initial age  (days) | Initial weight  (kg) | No. of weight recordings |
| --- | --- | --- | --- | --- | --- | --- | --- | --- | --- | --- |
| 1 | 2011 | Heifers | 83 | Individual pen | Canister | 1.623 ± 0.08 | 23 | 294 ± 26 | 219 ± 28 | 4 |
| 2 | 2011 | Bulls | 71 | Individual pen | Canister | 1.405 ± 0.05 | 23 | 268 ± 24 | 254 ± 34 | 19 |
| 3 | 2012 | Bulls | 90 | Individual pen | Canister | 2.334 ± 0.19 | 24 | 264 ± 23 | 229 ± 34 | 13 |
| 4 | 2012 | Heifers | 85 | Individual pen | Canister | 1.938 ± 0.16 | 25 | 325 ± 26 | 261 ± 28 | 14 |
| 5 | 2018 | Bulls | 83 | GrowSafe® | Cylinder | 3.119 ± 0.27 | 34 | 347 ± 28 | 270 ± 46 | 6 |
| 6 | 2018 | Bulls | 83 | GrowSafe® | Cylinder | 3.145 ± 0.23 | 36 | 354 ± 25 | 275 ± 43 | 6 |
| 7 | 2019 | Bulls | 83 | GrowSafe® | Cylinder | 4.549 ± 0.30 | 60 | 249 ± 31 | 224 ± 33 | 6 |
| 8 | 2019 | Bulls | 56 | Intergado® | Cylinder | 3.471 ± 0.17 | 58 | 647 ± 36 | 465 ± 39 | 2 |
| 9 | 2019 | Bulls | 56 | Intergado® | Cylinder | 3.062 ± 0.09 | 58 | 667 ± 35 | 573 ± 48 | 2 |
| 10 | 2019 | Bulls | 83 | GrowSafe® | Cylinder | 2.471 ± 0.15 | 62 | 329 ± 24 | 285 ± 49 | 7 |
| 11 | 2020 | Bulls | 83 | GrowSafe® | Cylinder | 2.621 ± 0.35 | 42 | 237 ± 24 | 226 ± 42 | 7 |
| 12 | 2020 | Bulls | 83 | GrowSafe® | Cylinder | 2.656 ± 0.34 | 44 | 239 ± 22 | 221 ± 33 | 7 |
