# Supplementary material for: Phenotypic association among performance, feed efficiency and methane emission traits in Nellore cattle
Source: PLoS One. 2021 Oct 14;16(10):e0257964. doi: 10.1371/journal.pone.0257964 (PMC8516271; doi:10.1371/journal.pone.0257964)
Supplement: S2 Table — (DOCX) [file pone.0257964.s002.docx]

**S2 Table**. **Percentage of ingredients and nutrient composition of diets offered to the animals during the performance test according each test group**

| Ingredients  (% DM) | Year of performance test | | | | | |
| --- | --- | --- | --- | --- | --- | --- |
|  | 2011 | 2012 | 2018 | 2019 | 2019 | 2020 |
| Corn silage | - | 53.6 | 54.0 | - | 27.6 | 60.0 |
| Sorghum silage | - | - | - | 60.0 | - | - |
| *Brachiaria* hay | 44.5 | 10.1 | - | - | - | - |
| Sugar cane bagasse | - | - | 10.2 | - | 4.89 | - |
| Cottonseed meal | 21.4 | - | - | - | - | - |
| Soybean meal | - | 11.6 | 11.7 | 13.0 | - | 13.0 |
| Peanut meal | - | - | - | - | 8.01 | - |
| Ground corn | 32.2 | 21.7 | 21.9 | 25.0 | - | 25.0 |
| Wet corn | - | - | - | - | 44.6 | - |
| Citrus pulp | - | - | - | - | 11.9 | - |
| Mineral premix | - | - | - | - | 1.78 | - |
| Salt | 1.45 | 2.28 | 1.70 | 1.75 | - | 1.75 |
| Ammonium sulfate | - | 0.072 | - | - | - | - |
| Urea | 0.45 | 0.648 | 0.49 | 0.25 | 1.16 | 0.25 |
| Forage to concentrate ration | 65:45 | 65:45 | 60:40 | 60:40 | 50:50 | 60:40 |
| Nutrients | | | | | | |
| Dry matter, % | 87.4 | 54.4 | 60.5 | 52.4 | 60.0 | 52.9 |
| Crude protein, % DM | 11.3 | 13.9 | 10.6 | 11.2 | 15.6 | 10.6 |
| Ash, % DM | 3.74 | - | 3.69 | 4.63 | - | 4.08 |
| Ether extract, % DM | 2.84 | 1.90 | 1.78 | 2.13 | 3.20 | 3.29 |
| Neutral detergent fiber, % DM | 50.0 | 50.2 | 48.1 | 40.6 | 26.9 | 35.6 |
| Acid detergent fiber, % DM | 31.0 | 22.9 | 30.7 | 24.4 | - | 21.3 |
| Gross energy, Mcal/kg | 4.09 | 4.16 | 3.73 | 3.77 | 4.11 | 4.47 |
| Non-fiber carbohydrates, DM% | 32.1 | 34.0 | 35.8 | 41.5 | 54.0 | 46.4 |
| Total digestible nutrients^1^, DM% | 70.5 | 70.2 | 65.9 | 70.2 | 77.0 | 75.1 |

^1^Values calculated using the equation of Weiss [24]. DM: dry matter. The diets were formulated for 0.800 kg/day in 2011 and 2012, for 1.200 kg/day in 2018, 2019 (60:40), and 2020, and for 1.700 kg/day in 2019 (50:50) [25].
