# Supplementary material for: Phenotypic association among performance, feed efficiency and methane emission traits in Nellore cattle
Source: PLoS One. 2021 Oct 14;16(10):e0257964. doi: 10.1371/journal.pone.0257964 (PMC8516271; doi:10.1371/journal.pone.0257964)
Supplement: S3 Table — (DOCX) [file pone.0257964.s003.docx]

**S3 Table**. **Mean values of performance, feed efficiency and enteric methane emission traits according to residual feed intake class of Nellore (*Bos indicus*)**

| Trait | N | Negative RFI  (n=246) | Positive RFI  (n=243) | SEM | P |
| --- | --- | --- | --- | --- | --- |
| Initial age (days) | 489 | 390 | 389 | 44.0 | 0.5353 |
| Initial body weight (kg) | 489 | 317 | 317 | 34.2 | 0.8675 |
| Mid-test body weight (kg) | 489 | 353 | 354 | 11.2 | 0.8498 |
| Dry matter intake (kg/day) | 489 | 7.405 | 8.550 | 0.23 | <0.0001 |
| Average daily gain (kg/day) | 489 | 1.228 | 1.237 | 0.07 | 0.7121 |
| Metabolic body weight (kg) | 489 | 79.7 | 79.8 | 1.59 | 0.8937 |
| RFI (kg/day) | 489 | -0.556 | 0.565 | 0.03 | <0.0001 |
| Feed conversion (kg/kg) | 489 | 6.695 | 7.764 | 0.453 | <0.0001 |
| Residual average daily gain (kg/day) | 489 | 0.066 | -0.064 | 0.014 | <0.0001 |
| CH_4_ (g/day) | 481 | 179.7 | 189.8 | 10.1 | 0.0022 |
| CH_4_/DMI (g/kg/day) | 481 | 23.46 | 21.34 | 1.09 | <0.0001 |
| CH_4_/ADG (g/kg/day) | 481 | 169.3 | 175.2 | 16.2 | 0.0724 |
| CH_4_/MBW (g/kg) | 481 | 0.529 | 0.548 | 0.03 | 0.0096 |
| CH_4_/BW^0.75^ (g/kg) | 481 | 2.259 | 2.353 | 0.14 | 0.0033 |
| CH_4_Res (g/day) | 481 | 4.811 | -4.953 | 1.95 | 0.0004 |
| CH_4_/GE (%GE) | 481 | 7.78 | 7.08 | 0.41 | <0.0001 |

RFI: residual feed intake; SEM: standard error of the mean; CH_4_: enteric methane emission; CH_4_/DMI: CH_4_ emission expressed per dry matter intake; CH_4_/ADG: CH_4_ emission expressed per average daily gain, CH_4_/MBW: CH_4_ emission expressed per mid-test body weight; CH_4_/BW^0.75^ = CH_4_ emission expressed per metabolic body weight; CH_4_Res: residual CH_4_ emission; CH_4_/GE: % consumed gross energy lost as CH_4_.
