# Supplementary material for: Phenotypic association among performance, feed efficiency and methane emission traits in Nellore cattle
Source: PLoS One. 2021 Oct 14;16(10):e0257964. doi: 10.1371/journal.pone.0257964 (PMC8516271; doi:10.1371/journal.pone.0257964)
Supplement: S4 Table — (DOCX) [file pone.0257964.s004.docx]

**S4 Table.** **Studies in the literature showing the relationship between residual feed intake classes and enteric methane emission**

| Reference | N | Sex category | Cattle breed | Measurement technique | CH_4_  (g/day) | | *P* | CH_4_/DMI (g/kg/day) | | *P* |
| --- | --- | --- | --- | --- | --- | --- | --- | --- | --- | --- |
|  |  |  |  |  | RFI- | RFI+ |  | RFI- | RFI+ |  |
| Nkrumah et al. [13] | 19 | Steers | Continental x British | Indirect calorimetry | 135 | 180 | *<0.05* | 14.0 | 15.5 | *-* |
| Hegarty et al. [10] | 20 | Steers | Angus | SF_6_ | 142 | 190 | *0.01* | 16.3 | 14.7 | *0.37* |
| Jones et al. [14] | 25  48 | Pregnant cows  Cows | Angus | OP-FTIR | 133  182 | 125  227 | *-*  *<0.05* | 13.0  13.9 | 11.7  16.2 | *-*  *-* |
| Fitzsimons et al. [33] | 14 | Heifers | Simmental | SF_6_ | 260 | 297 | *0.04* | 38.0 | 36.0 | *0.52* |
| Sharma et al. [34] | 6 | Calves | Sahiwal | SF_6_ | 58.7 | 65.6 | *<0.05* | 15.3 | 18.9 | *<0.05* |
| McDonnell et al. [35] | 28 | Heifers | Limousin x Friesian | SF_6_ | 156 | 146 | *0.11* | 22.4 | 20.2 | *0.034* |
| Alemu et al. [36] | 16 | Heifers | Crossbred | GreenFeed  Respirometry chamber | 203  156 | 222  165 | *0.02*  *0.40* | 27.7  26.5 | 28.5  26.5 | *0.25*  *0.99* |
| Dini et al. [30] | 16 | Steers | Hereford | SF_6_ | 194 | 265 | *0.009* | 20.3 | 28.1 | *0.021* |
| Flay et al. [32] | 56 | Heifers | Jersey/Holstein-Friesian | GreenFeed | 253 | 256 | *0.60* | 22.7 | 20.7 | *<0.01* |
| Manafiazar et al. [31] | 314  139 | Heifers  Cows | Crossbred | GreenFeed | 180  233 | 184  241 | *0.001*  *<0.001* | 24.1  21.1 | 22.7  19.2 | *<0.001*  *<0.001* |
| Batalha et al. [19] | 24 | Bulls | Nellore | SF_6_ | 235 | 249 | *0.365* | 25.3 | 26.2 | *0.389* |

SF_6_: SF_6_ tracer gas technique; OP-FTIR: open-path Fourier transform infrared spectroscopy; CH_4_: methane emission; RFI-: negative residual feed intake.; RFI+: positive residual feed intake.
